# Extraction, Structural Characterization, and Anti-Hepatocellular Carcinoma Activity of Polysaccharides From *Panax ginseng* Meyer

**DOI:** 10.3389/fonc.2021.785455

**Published:** 2021-11-29

**Authors:** Hui Jia, Bin Zhao, Fangfang Zhang, Ramesh Kumar Santhanam, Xinying Wang, Jincai Lu

**Affiliations:** ^1^ School of Traditional Chinese Materia Medica, Shenyang Pharmaceutical University, Shenyang, China; ^2^ Department of Stomatology, The Fifth Medical Center of People’s Liberation Army (PLA) General Hospital, Beijing, China; ^3^ Faculty of Science and Marine Environment, Universiti Malaysia Terengganu, Kuala Nerus, Malaysia; ^4^ Liaoning Provincial Key Laboratory of Traditional Chinese Medicine (TCM) Resources Conservation and Development, Shenyang Pharmaceutical University, Shenyang, China

**Keywords:** *Panax ginseng* Meyer, MCG polysaccharides, NMR, HCC, GC–MS analysis

## Abstract

Polysaccharides are the main active ingredients of ginseng. To extract the most effective polysaccharides against hepatocellular carcinoma (HCC), we isolated and characterized the polysaccharides from the mountain cultivated ginseng (MCG) and compared their composition and cytotoxic effect with cultivated ginseng (CG) polysaccharide against HepG2 cell lines for the first time. MCG polysaccharides and CG polysaccharides were fractionated into two fractions such as MTPS-1, MTPS-2 and CTPS-1, CTPS-2 by salting out, respectively. Compared to CG, MCG possessed appreciable cytotoxic effect against HepG2 cells among that MTPS-1 possess fortified effect. Then, MTPS-1 was selected for further isolation process and seven acidic polysaccharides (MCGP-1–MCGP-7) were obtained using ethanol precipitation, ion-exchange, and gel permeation chromatography techniques. Structural characteristics of the polysaccharides (MCGP-1–MCGP-7) were done by adapting methylation/GC-MS and NMR analysis. Overall, MCGP-3 polysaccharide was found to possess significant cytotoxic effect against HepG2 cells with the IC_50_ value.

## 1 Introduction

Hepatocellular carcinoma (HCC) is one of the malignant tumors with poor prognosis and lower survival rate (1, 2). Among them, HCC accounts for more than 90% of liver cancer and has the characteristics of uncertain growth, invasion, and anti-apoptosis, which leads to drug resistance and treatment failure (3, 4). Therefore, it is still necessary to discover new and effective therapeutic drugs.

*Panax ginseng* Meyer is a well-known traditional Chinese medicine where the main components of this species are saponins and polysaccharides. The research on ginsenosides is relatively in-depth, however due to the molecular weight limitation, the studies on ginseng polysaccharides were limited. Nowadays, natural polysaccharides seek much attention among researchers due to their effectiveness and non-toxic nature (5–9). Recently polysaccharides from natural sources have been utilized as a drug to treat specific cancer owing to their substantial proliferation, migration, and apoptosis effect (10, 11).

Ginseng in the market is mainly classified into two grades: cultivated ginseng (CG) and mountain cultivated ginseng (MCG) (12). It has been pointed out in the literature that butanol-extracted wild ginseng (BX-MG) shows high anti-tumor activity by inhibiting the proliferation of HCC cells and inducing their apoptosis, which may be related to the presence of more saponins (13). However, there are no reports on the extraction and separation of MCG polysaccharides, structural analysis, and screening for anti-tumor activity. In this study, the monosaccharide composition and structure analysis of the polysaccharides from MCG were studied for the first time, and the anti-HCC activity of MCG polysaccharides was screened. In addition, the anti-HCC activity of MCG polysaccharide and CG polysaccharide were compared, and the relationship between the activity and structure of ginseng polysaccharide were briefly discussed.

## 2 Material and Methods

### 2.1 Materials

The roots of cultivated ginseng (5 years old) were collected from the Changbai Mountain, Huanren, Liaoning Province, China in February 2019. Mountain cultivated ginseng (MCG) roots were 15 years old, and the plants were grown at the Changbai Mountain, Huanren, Liaoning Province. 1-Phenyl-3-methyl-5-pyrazalone (PMP) was obtained from the Shanghai Macklin Biochemical Co., Ltd. (China). Monosaccharide standards including galacturonic acid (GalA), glucuronic acid (GlcA), glucose (Glc), rhamnose (Rha), arabinose (Ara), galactose (Gal), xylose (Xyl), and mannose (Man) were purchased from Wako (Japan). Molecular Weight of Dextrans were purchased from the Beijing Century Aoke Biotechnology Co., Ltd. (China). DMEM (Hyclone, UT, USA), FBS (Hyclone, Utah, USA), SRB (Rhodamine B, Jiangnanjie, China).

### 2.2 Methods

#### 2.2.1 Extraction of Crude Polysaccharides

The dried roots of mountain-cultivated ginseng (2 kg) were decocted with distilled water (40 L) three times. All aqueous solutions were combined, concentrated under reduced pressure and precipitated by adding of 95% ethanol (4 volumes) at 4°C for 24 h to obtain crude mountain-cultivated ginseng polysaccharides (MTPS). MTPS was further deproteinated by using the Sevag method and de-starch with α-amylase. MTPS was prepared into 5% solution with 1.5 mol/L NaCl, stirred at 50°C for 4 h, and left at 4°C overnight (14). After centrifugation at 4,000 r/min for 10 min, the precipitation and supernatant were collected, respectively. The supernatant was dialyzed for 48 h and lyophilized to obtain salt-soluble polysaccharides (MTPS-1). The precipitation was dissolved with distilled water, dialyzed for 48 h and lyophilized to obtain salt-insoluble polysaccharides (MTPS-2). MTPS-1 was re-dissolved with distilled water then sub-fractionated by graded precipitation at final ethanol concentrations of 40, 50, 60, 70, and 80%, respectively. The five precipitation fractions of MCG polysaccharides were named MTPS-40, MTPS-50, MTPS-60, MTPS-70, and MTPS-80, respectively. MTPS-40 was further fractioned by a graded precipitation at different final ethanol concentrations, namely, 40, 60, and 80% (*v/v*), corresponding to the three sub-fractions assigned to MTPS-40 (I), MTPS-40 (II), and MTPS-40 (II).

**Figure 1:**
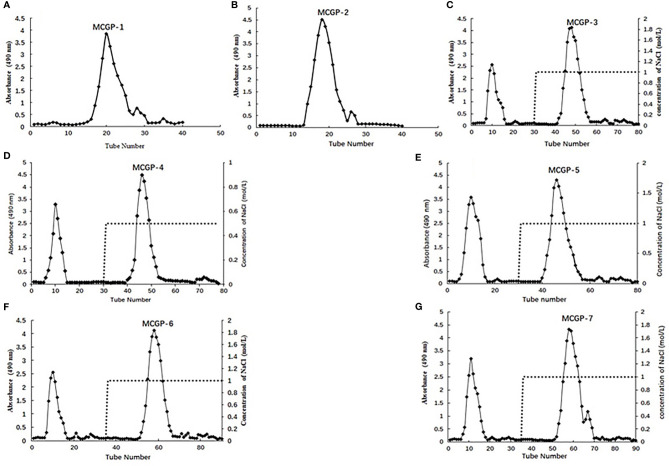
**(A, B)** Elution profiles of MCGP-1, MCGP-2 on a Sephadex G-25 column, **(C–G)** MCGP-3~MCGP-7 on DEAE-52 column, eluted with H_2_O and different concentration of NaCl, respectively.

The seven fractions were further purified by DEAE-52 column chromatography and gel column chromatography (**Figure 1**). The MCGP-1–MCGP-7 homogeneous polysaccharides were obtained from the seven fractions (MTPS-40 (I–II) to MTPS-80). Cultivated ginseng powder (200 g) was prepared in the same way as mountain-cultivated ginseng to obtain crude cultivated ginseng polysaccharide (CTPS), cultivated ginseng salt-soluble polysaccharides (CTPS-1), and cultivated ginseng salt-insoluble polysaccharides (CTPS-2). The total sugar content was determined by the phenol-sulfuric acid method at 490 nm with the standard of D-glucose (15). The protein content was determined by Bradford method at 595 nm with the standard of bovine serum albumin (BSA) (16).

#### 2.2.2 Homogeneity, Molecular Weight and Monosaccharide Composition Determination

The homogeneity, molecular weight and monosaccharide composition analysis were performed according to the previous method (17).

#### 2.2.3 Partial Acidic Hydrolysis

MCGP-3 (50 mg) and MCGP-4 (50 mg) were treated with 0.1 M TFA (20 ml) at 90°C. After hydrolysis for 1, 2, 4, 6, and 8 h, the hydrolysate (3 ml) was taken and dialyzed against distilled water using dialysis membrane (MWCO 3,500 Da), respectively. The solution inside and outside of the dialysis membrane were evaporated to dryness under reduced pressure, hydrolyzed, and their monosaccharide composition was analyzed (18).

#### 2.2.4 Methylation and GC–MS Analysis

The method of Ciucanu and Kerek (19) was used for methylation analysis and the other details were referenced to the previous report (11). For the uronic acid-containing polysaccharide, the reduction of uronic acid was carried out according to the Feng method (20). Finally, after compared the linkages of native polysaccharide and reduced polysaccharide, the linkage of every residue was concluded.

#### 2.2.5 NMR Spectroscopic Analysis

MCGP-3 and MCGP-4 were exchanged with deuterium by freeze-drying D_2_O (99.9 atom%) three times. The samples (20 mg) were finally dissolved in D_2_O. The ^13^C NMR (150 MHz) and heteronuclear single quantum coherence (HSQC) spectra were obtained on a Brucker Avance III HD 600 spectrometer (Germany).

#### 2.2.6 Cell Viability Assay

HepG2 cells were purchased from American Type Culture Collection (ATCC, Manassas, VA, USA). HepG2 cells were cultured in DMEM medium containing 10% fetal bovine serum (FBS) and 1% penicillin–streptomycin and placed in a 37°C incubator filled with 5% CO_2_ gas (21).

The activity screening of polysaccharides were determined using Sulforhodamine B (SRB) experiment (22). HepG2 cells was seeded into 96-well plates and treated with different concentrations of monomer compound/mixture for 48 h. After drug treatment, Cells were fixed with 50% TCA solution. The cells were stained with 0.4% SRB staining solution, and the optical density (OD) value was measured at 540 nm using a microplate reader (Elx 800 Bio-Tek, USA). Experiments were repeated at least three times.

#### 2.2.7 Statistical Analysis

The data were displayed as means ± SEM. Statistical analysis was performed using GraphPad Prism 7.0 (SanDiego, CA, USA). To compare the differences between multiple groups, one-way analysis of variance (ANOVA) was performed in this software. *P <*0.05 indicated statistically significant.

## 3 Results

### 3.1 Extraction, Separation and Purification of *P. ginseng* Polysaccharide

**Figure 2:**
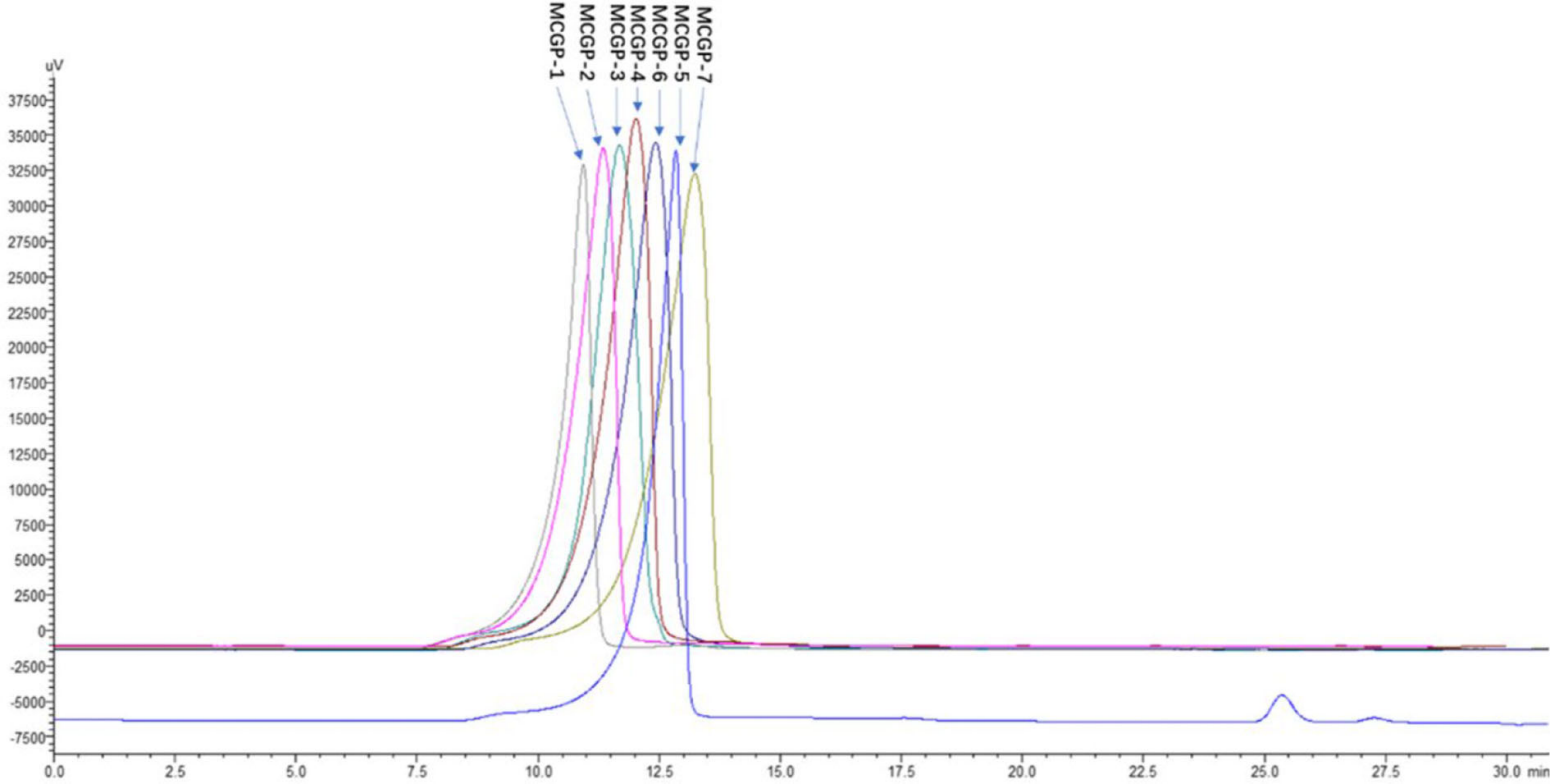
HPGPC elution profiles of MCGP-1–MCGP-7. Elution profile of polysaccharides (MCGP-1–MCGP-7) from *P. ginseng* using HPGPC with a refractive index (RI) detector.

The roots of MCG was decocted with distilled water and precipitated by alcohol, de-starch with α-amylase and deproteinized by Sevag method to obtain crude mountain-cultivated ginseng polysaccharides (MTPS). The MTPS was divided into salt soluble polysaccharide (MTPS-1) and salt insoluble polysaccharide (MTPS-2) by salting out. CG polysaccharides (CTPS) was prepared in the same way as MTPS, CTPS was divided into cultivated ginseng salt-soluble polysaccharides (CTPS-1) and cultivated ginseng salt-insoluble polysaccharides (CTPS-2) by salting out. MTPS-I was re-dissolved with distilled water then fractionated by graded precipitation at final ethanol concentrations of 40, 50, 60, 70, and 80%, respectively. The five fractions were further purified by DEAE-52 column chromatography and gel column chromatography to obtain seven homogeneous polysaccharides MCGP-1–MCGP-7. MCGP-1–MCGP-7 showed a single symmetric peak by HPGPC (**Figure 2**).

### 3.2 Monosaccharide Composition and Molecular Weight Distribution Analysis

The molecular weight, protein content, total sugar content and monosaccharide compositions of all the fractions from the CG and MCG are shown in **Table 1**. MCGP-1–MCGP-7 were mainly composed of GalA, Ara, Gal, Rha, and Glc. In addition, they contained small amounts of Man and GlcA. The ratios of Rha/GalA for MCGP-1, MCGP-3, MCGP-4, and MCGP-5 were 0.82, 0.38, 0.17, and 0.24, respectively, which are among the rhamnogalacturonan I (RG-I) range from 0.05 to 1.0 defined by Schols and Voragen (23). The average molecular weight was estimated in reference to the calibration curve of standard dextran. A calibration curve generated using the Log MW of the standard *versus* their retention time (RT) was obtained (Log MW = −0.5 RT + 9.5592, *r* ^2^ = 0.9962).

**Table 1:** Monosaccharide composition of MCGP-1–MCGP-7.

Samples	Mw (kDa)	Protein content (μg/mg)	Total Sugar (%)	Sugar components (%)							
				Glc	Gal	GlcA	GalA	Rha	Man	Ara	Xyl
MTPS	1.734 × 10^5^–3.04 × 10^3^	13.70	89.1%	62.32	16.14	0.331	7.44	2.45	1.675	9.645	n.d.
MTPS-1	1.783 × 10^5^–2.50 × 10^3^	6.62	90.9%	22.11	23.12	0.534	18.17	4.323	1.493	27.02	0.673
MTPS-2	1.740 × 10^5^–2.56 × 10^3^	3.540	75.0%	35.92	19.76	0.739	17.99	4.64	0.925	19.55	0.312
CTPS	1.787 × 10^5^–2.21 × 10^3^	15.88	87.5%	46.24	21.26	1.37	10.70	3.23	1.35	15.85	n.d.
CTPS-1	1.781 × 10^5^–2.58 × 10^3^	12.47	89.6%	38.06	23.90	0.641	12.80	4.09	1.747	18.78	n.d.
CTPS-2	1.730 × 10^5^–2.43 × 10^3^	12.24	77.5%	34.64	20.62	0.427	18.57	3.389	1.106	21.24	n.d.
MCGP-1	1.649 × 10^5^	5.62	–	5.686	44.49	0.5460	8.011	6.577	0.2680	34.42	n.d.
MCGP-2	1.644 × 10^5^	n.d.	–	9.132	46.77	0.6060	6.383	6.480	0.3810	27.88	n.d.
MCGP-3	1.572 × 10^5^	6.12	–	33.17	22.88	0.6870	15.67	6.005	0.6310	20.96	n.d.
MCGP-4	1.673 × 10^5^	1.52	–	7.146	39.74	1.519	26.74	4.533	0.2140	20.11	n.d.
MCGP-5	1.60 × 10^5^	1.02	–	5.748	39.36	1.728	16.85	4.092	2.784	25.60	n.d.
MCGP-6	1.592 × 10^5^	n.d.	–	1.764	45.99	1.233	3.722	7.295	0.3300	39.66	n.d.
MCGP-7	1.52 × 10^5^	n.d.	–	1.646	17.98	0.6570	2.830	9.456	0.3420	67.09	n.d

n.d., not detected.

### 3.3 Methylation Analysis

Methylation analysis was performed to determine the linkage pattern of MCGP-3 and MCGP-4. The procedure involved derivation of the monosaccharide component of MCGP-3–MCGP-4 to PMAAs, which were analyzed by GC–MS. There were six types of PMAAs, namely, 2,3,4,6-Me_4_-Glc*p*, 2,3,6-Me_3_-Glc*p*, 2,3,4,6-Me_4_-Gal*p*, 2,3,6-Me_3_-Gal*p*, 3,4-Me_2_-Rha*p*, 2,3,5-Me_3_-Ara*f*, 2,3-Me_2_-Ara*f*, 2-Me-Ara*f*, 2,3,4-Me_3_-Gal*p*, and 2,4,5-Me_4_-Gal*p*; which were assigned to Glc*p*-(1→, →4)-Glc*p*-(1→, Gal*p*-(1→, →4)-Gal*p*-(1→, →2)-Rha*p*-(1→, Ara*f*-(1→, →5)-Ara*f*-(1→, →3,5)-Ara*f*-(1→, →6)-Gal*p*-(1→ and →3,6)-Gal*p*-(1→ residues, respectively.

Methylation results demonstrated that MCGP-3 mainly consisted of 1→linked Glc*p* (12.5%), (1→4)-linked Glc*p* (27.6%), 1→linked Gal*p* (6.3%), (1→4)-linked Gal*p* (12.8%), (1→2)-Rha*p* (6.53%), 1→linked Ara*f* (12.9%), (1→5)-linked Ara*f* (15.7%), and (1→3, 5)-linked Ara*f* (13.2%). MCGP-4 mainly consisted of (1→4)-linked Glc*p* (6.8%), (1→6)-linked Gal*p* (15.6%), (1→4)-linked Gal*p* (10.92%), (1→3, 6)-linked Gal*p* (15.27%), (1→2)-linked Rha*p* (9.72%), 1→linked Ara*f* (12.76%), (1→5)-linked Ara*f* (14.12%), and (1→3, 5)-linked Ara*f* (9.27%), as shown in **Table 2**. Compared to MCGP-3 and MCGP-4, the carboxyl-reduced MCGP-3-R and MCGP-4-R showed an increase ratio of 1, 4-linked galactose, indicating that the galacturonic acid in MCGP-3 and MCGP-4 was 1, 4-linked.

**Table 2:** Results of methylation analysis of MCGP-3 and MCGP-4.

Methylated sugars	Type of linkage	MCGP-3 (%)	MCGP-4 (%)
2,3,4,6-Me_4_-Glc*p*	Glc*p*-(1→	12.5	2.15
2,3,6-Me_3_-Glc*p*	→4)-Glc*p*-(1→	27.6	6.8
2,3,4,6-Me_4_-Gal*p*	Gal*p*-(1→	6.3	–
2,3,4-Me_3_-Gal*p*	→6)-Gal*p*-(1→	–	15.6
2,3,6-Me_3_-Gal*p*	→4)-Gal*p*-(1→	12.8	10.92
2,4,5-Me_4_-Gal*p*	→3,6)-Gal*p*-(1→	–	15.27
3,4-Me_2_-Rha*p*	→2)-Rha*p*-(1→	6.53	9.72
2,3,5-Me_3_-Ara*f*	Ara*f*-(1→	12.9	12.76
2,3-Me_2_-Ara*f*	→5)-Ara*f*-(1→	15.7	14.12
2-Me-Ara*f*	→3,5)-Ara*f*-(1→	13.2	9.27

### 3.4 Partial Acid Hydrolysis of MCGP-3 and MCGP-4

The hydrolysates of MCGP-3 and MCGP-4 from different hydrolysis times were analyzed by HPGPC. MCGP-3 was gradually hydrolyzed with different times, and the molecular weight of the remaining polysaccharide hydrolyzed at 8 and 6 h resembles same pattern, indicating that the core structures of MCGP-3 were not broken by acid hydrolysis, however, the side chains were gradually cleaved.

To investigate the core structures of MCGP-3 and MCGP-4, the hydrolysates present after 1, 2, 4, 6, and 8 h hydrolysis reactions were collected and separated *via* dialysis into two fractions. Fraction I contained the intact core, found in the dialysis membrane, and fraction II contained oligosaccharides and monosaccharides, found in the dialysate. In the remaining polymer of MCGP-3, Rha and GalA increased from 6.005 and 15.67% (MCGP-3-0 h) to 9.848 and 42.751% (MCGP-3-8 h-I), respectively; Ara and Gal decreased from 20.956 and 22.877 to 0.901% and 9.573, respectively. In the remaining polymer of MCGP-4, Rha and GalA increased from 4.53 and 26.74% (MCGP-4-0 h) to 6.54 and 62.43% (MCGP-4-8 h-I), respectively; Ara and Gal decreased from 20.11 and 39.74 to 1.73 and 17.65%, respectively. The changes in monosaccharide composition of the released monosaccharides and oligosaccharides were found to be consistent with those observed for the remaining polymer. These changes in monosaccharide composition indicated that after 8 h of hydrolysis, Ara was completely cleaved, Gal was partially hydrolyzed, and little of Rha and GalA were hydrolyzed, indicating that Ara was located on the surface of the molecules, Rha and GalA probably exist in the core of MCGP-3 or MCGP-4.

### 3.5 NMR Spectroscopy Analysis

**Figure 3:**
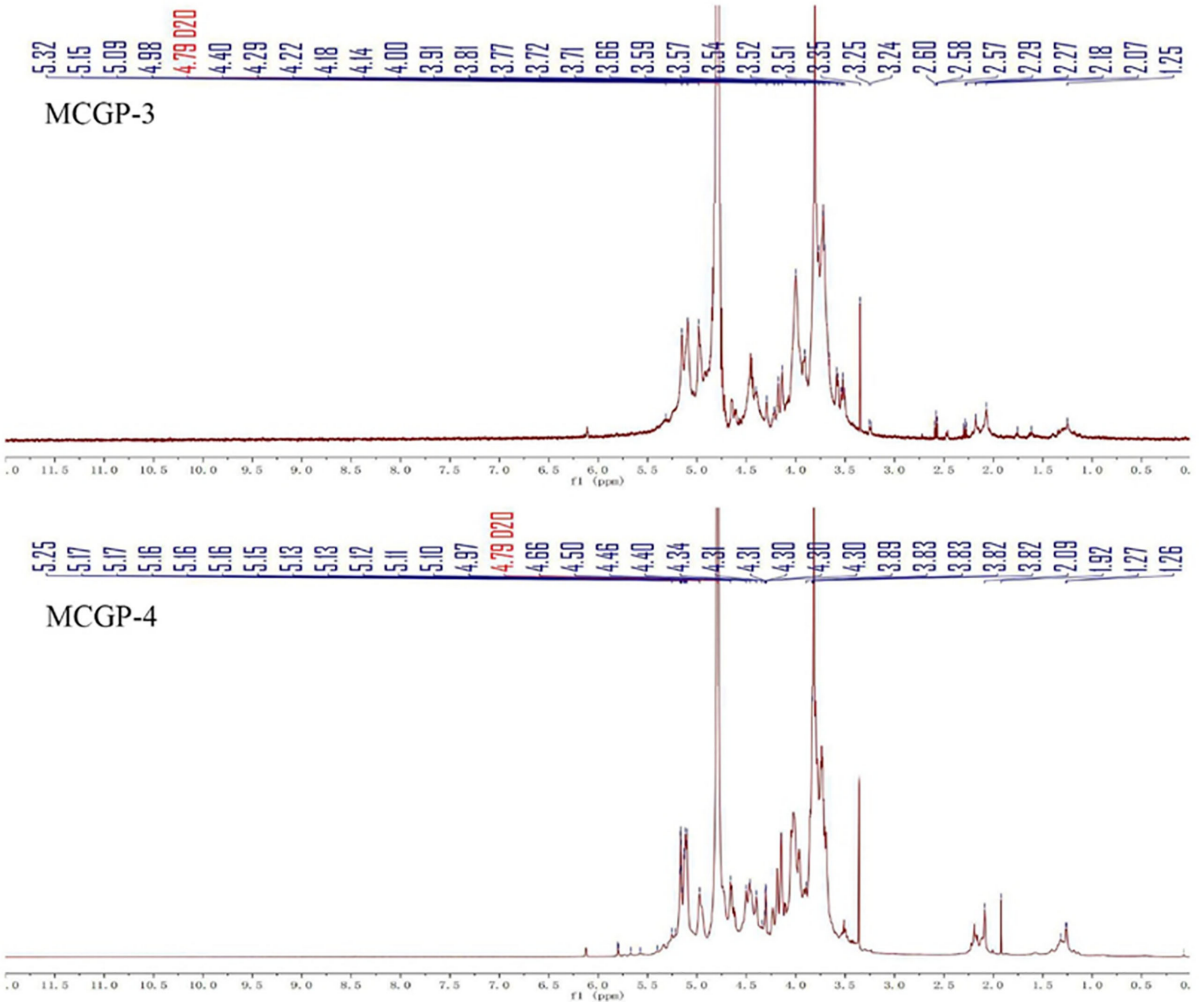
^1^H NMR spectra of MCGP-3 and MCGP-4.

**Figure 4:**
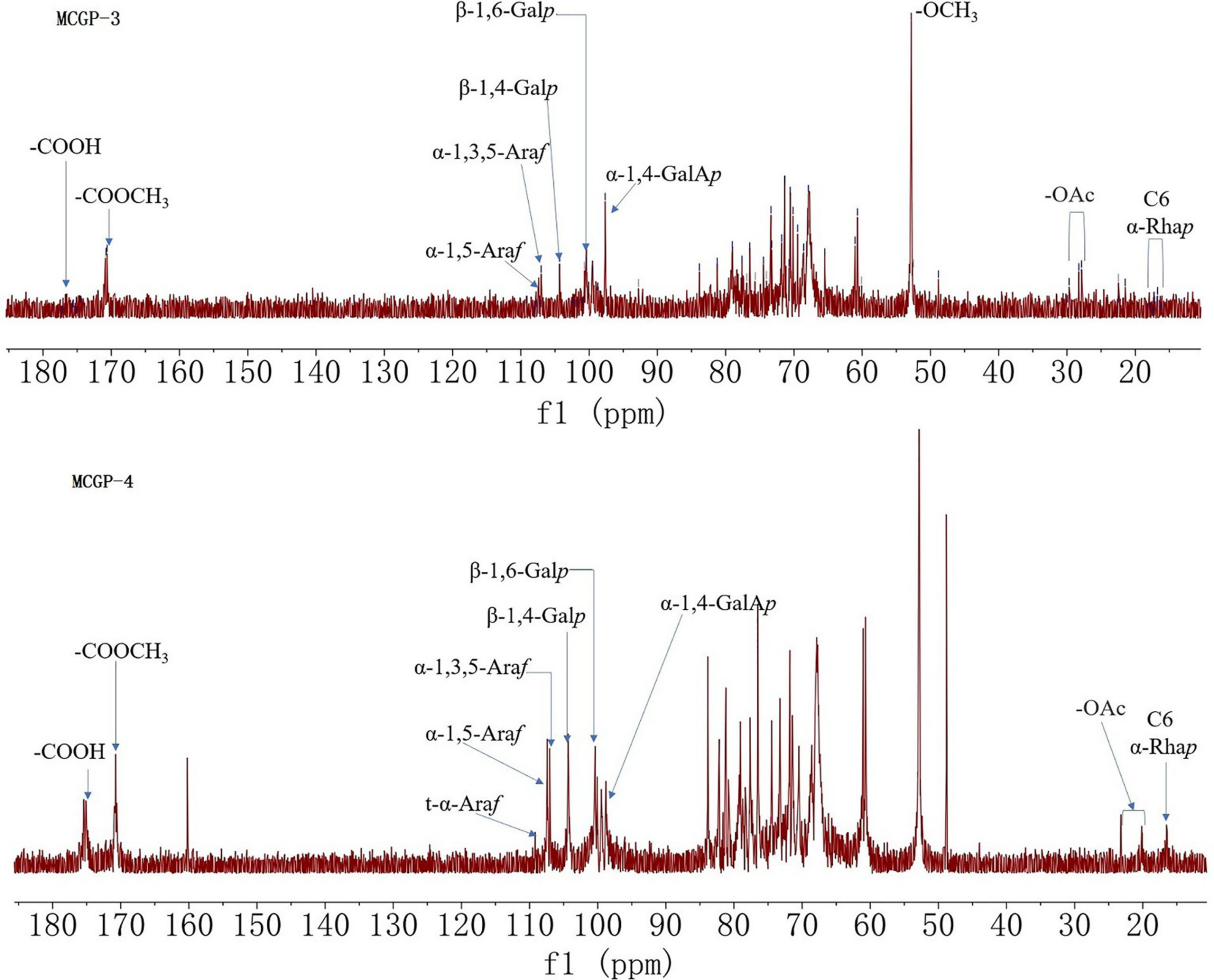
^13^C NMR spectra of MCGP-3 and MCGP-4.

**Figure 5:**
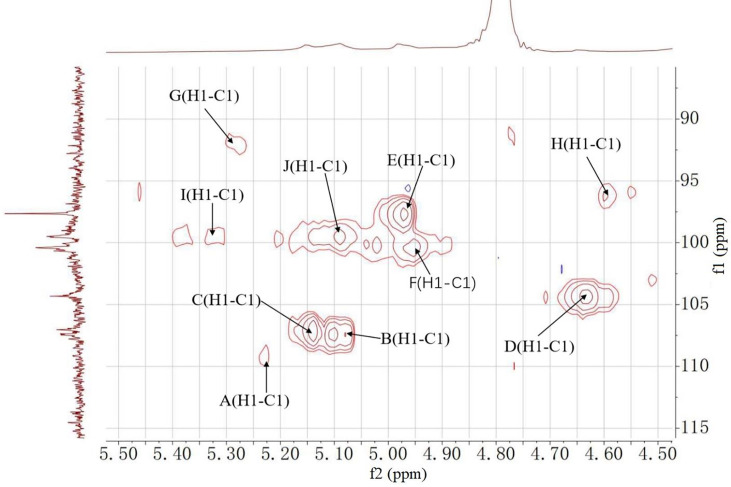
HSQC spectrum of MCGP-3.

^1^H, ^13^C NMR, and 2D NMR (HSQC) spectra were used to confirm the structure of MCGP-3 and MCGP-4. The NMR spectra were calibrated by D_2_O and the chemical shift of D_2_O was 4.79 ppm. From the ^1^H NMR spectrum of MCGP-3, the anomeric protons signals at 5.32, 5.28, 5.23, 5.14, 5.09, 5.08, 4.97, 4.95, 4.63, and 4.60 ppm were observed in ^1^H NMR spectrum (**Figure 3**). In the ^13^C NMR spectrum of MCGP-3 (**Figure 4**), anomeric carbon signals occurred at 109.09, 107.52, 107.07, 104.37, 100.32, 97.65, 92.22, 96.27, 99.50, and 99.65 ppm. The cross-peaks of 109.09/5.23, 107.52/5.08, 107.07/5.14, 104.37/4.63, 97.65/4.97, 100.32/4.95, 92.22/5.28, 96.27/4.60, 99.50/5.32, 99.65/5.09, and 99.65/5.09 ppm were observed in HSQC spectrum of MCGP-3 (**Figure 5**), which were assigned to residues of A, B, C, D, E, F, G, H, I, and J, respectively. The spectra of MCGP-3 were partially assigned on the basis of the HSQC experiment and by comparison with previous studies. The signals at H-1/C-1 109.09/5.23, 107.52/5.08, and 107.07/5.14 ppm were assigned to t-α-Ara*f*, →5)-α-Ara*f*-(1→ and →3, 5)-α-Ara*f*-(1→, respectively (5, 18). The signals at H-1/C-1 104.37/4.63 and 100.32/4.95 ppm were assigned to →4)-β-Gal*p*-(1→ and α-Gal*p-*(1→, respectively (24, 25). Similarly, the signals at H-1/C-1 92.22/5.28, 96.27/4.60 and 99.50/5.32 ppm were assigned to →4)-α-Glc*p*, β-D-Glc*p*-(1→ and →4)-α-D-Glc*p*-(1→, respectively (5, 26). The signals at 97.65 and 17.36 ppm were assigned to C-1 and C-6 of →2)-Rha*p*-(1→, while the corresponding chemical shift in the anomeric protons was δ 4.97 ppm (27). The signals for C-1-C-5 of esterified GalA residues did not give obvious signals because of the low degree of esterification, while the methyl group and methyl ester carbonyl carbons showed the diagnostic signals at 52.81 and 170.75 ppm, respectively (28). Taking the results of methylation analysis and partial acid hydrolysis into consideration, the backbone of MCGP-3 was composed of →4)-α-D-Gal*p*A-(1→, →4)-α-D-Glc*p*-(1→ and a small amount of →2)-Rha*p*-(1→ and →4)-α-D-Gal*p*-(1→, substituted by different types of branches attached to *O*-6 of (1→4)-α-D-Glc*p* and *O*-5 of →3)-α-Ara*f*-(1→. The branches were mainly composed of terminal α-L-Ara-(1→, (1→4)-β-D-Gal and (1→5)-α-L-Ara.

As shown in ^13^C NMR, MCGP-3, and MCGP-4 have similar monosaccharide residues. Except for the monosaccharide residue in MCGP-3, there were 1,6-Gal*p* residue signals in the ^13^C NMR spectrum of MCGP-4, the six signals were at 102.95, 71.78, 73.44, 73.25, 70.08, and 68.69 ppm (24). In the ^13^C NMR spectrum of MCGP-4, the signals for C-1 and C-6 of unesterified GalA residues appeared at 99.25 (C-1) and 175.08 (C-6) ppm (29). Taking the results of methylation analysis into consideration, there was 1,3,6-Gal*p* residue in MCGP-4. The signals at δ 103.02, 71.42, 81.60, 70.49, 70.52, and 68.69 ppm were assigned to the C-1–C-6 of →3, 6)-α-D-Gal*p*-(1→ residue (30). Taking the results of methylation analysis and partial acid hydrolysis into consideration, the backbone of MCGP-4 was composed of →4)-α-D-GalA*p*-(1→, →4)-α-D-Gal*p*-(1→ or →6)-α-D-Gal*p*-(1→ and a small amount of →2)-Rha*p*-(1→ and →4)-α-D-Gal*p*-(1→, substituted by different types of branches attached to *O*-6 of (1→3)-α-D-Galp and *O*-5 of →3)-α-Ara*f*-(1→. The branches were mainly composed of terminal α-L-Ara-(1→, (1→4)-β-D-Gal or (1→6)-β-D-Gal and (1→5)-α-L-Ara.

### 3.6 Assay of Polysaccharides From *P. ginseng* on HepG2 Cells Viability

Several studies revealed the inhibitory effect of cultivated ginseng (CG) polysaccharide on HepG2 cells. However, the activity of mountain ginseng (MCG) polysaccharides against HepG2 cells has not been reported. SRB is used to screen the activity of MCG polysaccharide and CG polysaccharide on HepG2 cells. After 48 h of treatment of HepG2 cells, MCG (MTPS, MTPS-1, and MTPS-2) compared with CG (CTPS, CTPS-1, and CTPS-2), the cell inhibition rate of MCG increased significantly, and the inhibitory effect is concentration-dependent. The order of IC_50_ value from low to high is: MTPS-1>MTPS-2>MTPS>CTPS-1>CTPS-2>CTPS. The above results indicated that the inhibitory effect of MCG polysaccharide on HepG2 cells is better than that of CG polysaccharide. Among them, the activity of MTPS-1 is the most prominent.

**Figure 6:**
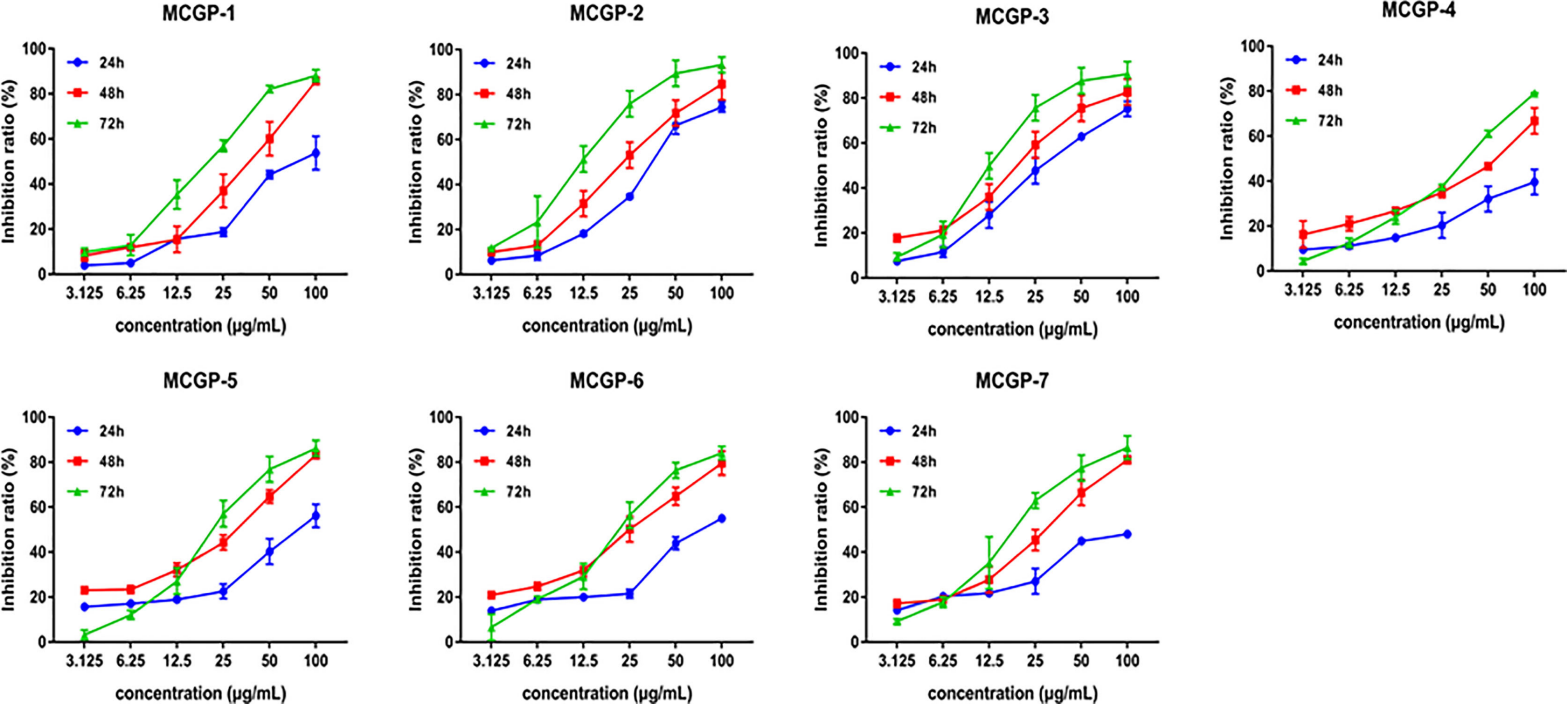
Effects of polysaccharides MCGP-1–7 from *Panax ginseng* on proliferation of HepG2 cells. Monomer components MCGP-1–7 are separated and purified from MCG. After treated HepG2 cells for 24, 48, and 72 h, the inhibition rate of MCGP-1–7 in HepG2 cells was measured by SRB. Line graph comparing the inhibition rates. Each value is the average ( ± SEM) of triplicate samples.

In order to explore the structure-activity relationship of polysaccharides, MTPS-1 were further fractionated into seven acidic fractions (MCGP-1–MCGP-7) by a combination of ethanol precipitation, ion-exchange and gel permeation chromatography. To further evaluate the inhibitory activity of 7 monomer compounds on HepG2 cells, the cells were treated with MCGP-1–MCGP-7 at different concentrations (3.125–100 μg/ml) for 24, 48, and 72 h, respectively. The result is shown in **Figure 6** and **Table 3**, MCGP-1–MCGP-7 at the concentration range of 3.125–50.0 µg/ml, significantly decreased the viability in HepG2 cells, and the inhibitory effect appears in a time and concentration dependent manner. After treatment for 72 h, MCGP-2 and MCGP-3 showed maximum cytotoxicity to HepG2 cells than others, with the IC_50_ value of 13.87 ± 0.54 and 13.02 ± 0.36 μg/ml, respectively. MCGP-4 showed low cytotoxicity to HepG2 cells, with the IC_50_ value of 35.67 ± 0.35 μg/ml.

**Table 3:** IC_50_ value of ginseng polysaccharide *in vitro*.

Active fraction	IC50 value (μg/ml) 48 h	Polysaccharide monomer	IC50 value (μg/ml)		
			24 h	48 h	72 h
MTPS	91.57 ± 1.50	MCGP-1	41.93 ± 3.23	36.53 ± 2.34	18.99 ± 1.35
MTPS-1	68.42 ± 0.65	MCGP-2	28.71 ± 2.22	18.42 ± 1.36	13.87 ± 0.54
MTPS-2	70.43 ± 2.82	MCGP-3	27.25 ± 1.67	19.20 ± 2.98	13.02 ± 0.36
CTPS	113.83 ± 1.58	MCGP-4	43.78 ± 1.25	49.82 ± 1.76	35.67 ± 0.35
CTPS-1	92.67 ± 1.22	MCGP-5	45.25 ± 1.64	32.21 ± 3.55	19.76 ± 1.44
CTPS-2	103.40 ± 0.73	MCGP-6	41.16 ± 2.66	43.54 ± 1.32	20.78 ± 1.74
		MCGP-7	32.91 ± 3.23	31.29 ± 2.24	20.02 ± 2.64

## 4 Discussion

Among HCC cases, the proportion of patients accounts for approximately 88% (31), it is necessary to discover and develop highly effective and low-toxic drugs for the treatment of HCC. Since synthetic drugs have their inevitable shortcomings, the natural products isolated from traditional Chinese medicine have attracted the attention of researchers in recent years.

In this study, the anti-tumor activity of MCG polysaccharides (MTPS, MTPS-1, and MTPS-2) and CG polysaccharides (CTPS, CTPS-1, and CTPS-2) was analyzed and compared, and it was found that MTPS-1 could significantly inhibit proliferation on HepG2 cells. Subsequently, MTPS-1 was further tracked and separated to obtain seven pectin polysaccharides MCGP-1–MCGP-7, and screened for anti HepG2 cells activity. The results show that MCGP-1–MCGP-7 all have relatively good anti- HepG2 cells activity. After 72 h of treatment, the order of inhibition rate were MCGP-3>MCGP-2>MCGP-1>MCGP-5>MCGP-7>MCGP-6>MCGP-4 has the most significant inhibitory effect on HepG2 cells, while the anti-HepG2 cells activity of MCGP-4 is relatively weak. The inhibition rates of MCGP-3 and MCGP-4 are different, which may be related to the differences of structure, conformation and monosaccharide composition.

Results of the structural characterization of MCGP-3–MCGP-4 indicated that the characteristic compositions of RG-I pectin, that is Rha, GalA, Ara, and Gal (17). The monosaccharide composition of MCGP-3 and MCGP-4 was similar, mainly containing Gal, GalA, Ara, Glc, and Rha, and a small amount of GlcA and Man. The content of Glc in MCGP-3 is higher than that of MCGP-4, and the content of GalA in MCGP-4 is higher than that of MCGP-3. The composition of main chains and branches of MCGP-3 and MCGP-4 was analyzed by partial acid hydrolysis. The study found that the main chain of MCGP-3 was mainly composed of GalA, Gal, Glc, and Rha, the content is 42.75, 9.57, 34.11, and 9.85%, respectively; the main chain of MCGP-4 was mainly composed of GalA, Gal, Glc, and Rha, the content is 62.43, 17.65, 4.28, and 6.54%, respectively. The branch chain of MCGP-3 was mainly composed of Gal, Ara, Glc, Rha, and GalA, the content is 31.98, 34.62, 15.90, 8.23, and 26.78%, respectively; the branch chain of MCGP-4 was mainly composed of Ara, Gal, Glc, Rha, and GalA, the content is 44.05, 48.07, 2.16, 2.78, and 1.90%, respectively. These results confirmed that MCGP-3 and MCGP-4 were RG-I-type pectin branched with α-1, 5/1,3,5-arabinan and *β*-1,4-galactan side chains. The biological activities of pectin might be related to the RG-I domains (28, 32). Several recent literatures have reported that some RG-I domains of the pectin have great immuno-modulating activities (33). The Ara residues linked to the surface of the molecules played an important role in enhancing lymphocyte proliferation activity (18). The anti-HepG2 cells activities of MCGP-1–MCGP-7 might be related to the Ara residues linked to the surface of the polysaccharide. In addition, the structure of MCGP-3 also contains disaccharide [-(1, 4)-α-D-GalAp-(1, 2. -α-L-Rhap-], which could enhance the anti-cancer activity of MCGP-3.

HCC is usually asymptomatic or atypical in its early stages. When the patient feels obvious discomfort, most of the disease has entered the advanced stage. HCC is not sensitive to chemotherapy and radiotherapy. Common treatment strategies include surgical resection, liver transplantation, vascular intervention, and radiofrequency ablation. Missed the early surgical resection treatment period, and can only choose drug treatment. It is used for the treatment of hepatocellular carcinoma where cancer cells have specific receptors, mainly including epidermal growth factor receptor (EGFR) inhibitors, vascular endothelial growth factor receptor (VEGFR) antagonists, etc. Sorafenib was the only molecularly targeted drug approved for the treatment of advanced hepatocellular carcinoma in China. Traditional Chinese medicine treatment of hepatocellular carcinoma has attracted much attention, and many active ingredients of traditional Chinese medicine can also be used for targeted therapy of HCC.

## 5 Conclusion

In this study, the polysaccharides from mountain cultivated ginseng (MCG) were extracted, isolated and structurally characterized for the first time. Moreover, the isolated polysaccharides from MCG were tested for their cytotoxic effect against HepG2 cells compared with polysaccharides from CG. Results revealed that the homogeneous polysaccharides MCGP-1–MCGP-7 isolated from MTPS-1 have a good inhibitory effect on HepG2 cells, and they are time- and concentration-dependent. Therefore, MCG polysaccharide is expected to become a potential new drug for the treatment of HCC

## Data Availability Statement

The original contributions presented in the study are included in the article/supplementary material. Further inquiries can be directed to the corresponding author.

## Author Contributions

HJ and BZ: Investigation, Writing – original draft, Visualization. HJ and XW: Writing – review and editing, Supervision. RS: Writing and Analysis. HJ, BZ, and FZ: Supervision, Funding acquisition. JL: Writing – review and editing, Supervision, Funding acquisition, Project administration. FZ: Focused on and has outstanding contributions the revision of this article. XW has done a lot of work in the processing of research data and experiment development. All authors contributed to the article and approved the submitted version.

## Funding

This work was supported by the National Key R&D Program of China (2017YFC1702302) and the Liaoning Revitalization Talents Program (XLYC1902119).

## Conflict of Interest

The authors declare that the research was conducted in the absence of any commercial or financial relationships that could be construed as a potential conflict of interest.

## Publisher’s Note

All claims expressed in this article are solely those of the authors and do not necessarily represent those of their affiliated organizations, or those of the publisher, the editors and the reviewers. Any product that may be evaluated in this article, or claim that may be made by its manufacturer, is not guaranteed or endorsed by the publisher.

## References

[1] Gao J Zhao Y Wang C Ji H Yu J Liu C. A Novel Synthetic Chitosan Selenate (CS) Induces Apoptosis in A549 Lung Cancer Cells *via* the Fas/FasL Pathway. Int J Biol Macromol (2020) 158:689–97. doi: 10.1016/j.ijbiomac.2020.05.016 32387597

[2] Lin L Cheng K Xie Z Chen C Chen L Huang Y. Purification and Characterization a Polysaccharide From Hedyotis Diffusa and its Apoptosis Inducing Activity Toward Human Lung Cancer Cell Line A549. Int J Biol Macromol (2019) 122:64–71. doi: 10.1016/j.ijbiomac.2018.10.077 30342944

[3] Travis WD Brambilla E Nicholson AG Yatabe Y Austin JHM Beasley MB. The 2015 World Health Organization Classification of Lung Tumors: Impact of Genetic, Clinical and Radiologic Advances Since the 2004 Classification. J Thorac Oncol (2015) 10:1243–60. doi: 10.1097/JTO.0000000000000630 26291008

[4] Guessous I Cornuz J Paccaud F. Lung Cancer Screening: Current Situation and Perspective. Swiss Med Weekly (2007) 137:304–11. 10.4414/smw.2007.11582 17629808

[5] Li B Zhang N Feng Q Li H Wang D Ma L. The Core Structure Characterization and of Ginseng Neutral Polysaccharide With the Immune-Enhancing Activity. Int J Biol Macromol (2019) 123:713–22. doi: 10.1016/j.ijbiomac.2018.11.140 30458191

[6] Wang X Lin Z. Immunomodulating Effect of Ganoderma (Lingzhi) and Possible Mechanism. Adv Exp Med Biol (2019) 1182:1–37. doi: 10.1007/978-981-32-9421-9_1 31777013

[7] Gu J Zhang H Wen C Zhang J He Y Ma H. Purification, Characterization, Antioxidant and Immunological Activity of Polysaccharide From Sagittaria Sagittifolia L. Food Res Int (2020) 136:109345. doi: 10.1016/j.foodres.2020.109345 32846537

[8] Chaves PFP Adami ER Acco A Iacomini M Cordeiro LMC. Chemical Characterization of Polysaccharides From Baccharis Trimera (Less.) DC. Infusion and Its Hepatoprotective Effects. Food Res Int (2020) 136:109510. doi: 10.1016/j.foodres.2020.109510 32846588

[9] Zhang Y Wu Z Liu J Zheng Z Li Q Wang H. Identification of the Core Active Structure of a Dendrobium Officinale Polysaccharide and its Protective Effect Against Dextran Sulfate Sodium-Induced Colitis *via* Alleviating Gut Microbiota Dysbiosis. Food Res Int (2020) 137:109641. doi: 10.1016/j.foodres.2020.109641 33233220

[10] Liu Z Gao T Yang Y Meng F Zhan F Jiang Q. Anti-Cancer Activity of Porphyran and Carrageenan From Red Seaweeds. Molecules (Basel Switzerland) (2019) 24(23):4286. doi: 10.3390/molecules24234286 PMC6930528 31775255

[11] Zhao B Wang X Liu H Lv C Lu J. Structural Characterization and Antioxidant Activity of Oligosaccharides From Panax Ginseng C. A. Meyer. Int J Biol Macromol (2020) 150:737–45. doi: 10.1016/j.ijbiomac.2020.02.016 32027898

[12] Zhao B Wang XY Luo W Lin Y Lv CN Lu JC. Isolation and Structural Elucidation of a Low-Molecular-Weight Polysaccharide From the Roots of Panax Ginseng C. A. Meyer. Nat Prod Res (2020) 30:1–8. doi: 10.1080/14786419.2020.1788025 32603191

[13] Hwang JW Oh JH Yoo H-S Lee Y-W Cho C-K Kwon K-R. Mountain Ginseng Extract Exhibits Anti-Lung Cancer Activity by Inhibiting the Nuclear Translocation of NF-κB. Am J Chin Med (2012) 40:187–202. doi: 10.1142/S0192415X12500152 22298458

[14] Chen F Huang G. Antioxidant Activity of Polysaccharides From Different Sources of Ginseng. Int J Biol Macromol (2019) 125:906–8. doi: 10.1016/j.ijbiomac.2018.12.134 30572039

[15] Michel Dubois KAG Hamilton JK Rebers PA Smith F. Colorimetric Method for Determination of Sugars and Related Substances. Anal Chem (1956) 28:350–6. doi: 10.1021/ac60111a017

[16] Sedmak JJ Grossberg SE. A Rapid, Sensitive, and Versatile Assay for Protein Using Coomassie Brilliant Blue G250. Anal Biochem (1977) 79:544–52. doi: 10.1016/0003-2697(77)90428-6 68686

[17] Qu H Yang W Li J. Structural Characterization of a Polysaccharide From the Flower Buds of Tussilago Farfara, and its Effect on Proliferation and Apoptosis of A549 Human non-Small Lung Cancer Cell Line. Int J Biol Macromol (2018) 113:849–58. doi: 10.1016/j.ijbiomac.2018.03.005 29505876

[18] Zhang X Li S Sun L Ji L Zhu J Fan Y. Further Analysis of the Structure and Immunological Activity of an RG-I Type Pectin From Panax Ginseng. Carbohydr Polymers (2012) 89:519–25. doi: 10.1016/j.carbpol.2012.03.039 24750753

[19] Kerek ICAF. A Simple and Rapid Method for the Permethylation of Carbohydrates. Carbohydr Res (1984) 131:209–17. doi: 10.1016/0008-6215(84)85242-8

[20] Feng L Yin J Nie S Wan Y Xie M. Fractionation, Physicochemical Property and Immunological Activity of Polysaccharides From Cassia Obtusifolia. Int J Biol Macromol (2016) 91:946–53. doi: 10.1016/j.ijbiomac.2016.05.030 27177462

[21] Jia H Yang Q Wang T Cao Y Jiang Q-Y Ma H-D. Rhamnetin Induces Sensitization of Hepatocellular Carcinoma Cells to a Small Molecular Kinase Inhibitor or Chemotherapeutic Agents. Biochim Biophys Acta (2016) 1860:1417–30. doi: 10.1016/j.bbagen.2016.04.007 27091611

[22] Wang S Zhang Y Ren T Wu Q Lu H Qin X. A Novel 4-Aminoquinazoline Derivative, DHW-208, Suppresses the Growth of Human Breast Cancer Cells by Targeting the PI3K/AKT/mTOR Pathway. Cell Death Dis (2020) 11:491. doi: 10.1038/s41419-020-2690-y 32606352 PMC7327080

[23] Schols HA Voragen AGJ. Complex Pectins: Structure Elucidation Using Enzymes. Prog Biotechnol (1996) 14:3–19. doi: 10.1016/S0921-0423(96)80242-5

[24] Li J Deng Q Yu X Wang W. Structural Studies of a New Fraction Obtained by Gradient Ethanol Precipitation From Acacia Seyal Gum. Food Hydrocolloids (2020) 107:105932. doi: 10.1016/j.foodhyd.2020.105932

[25] Chan MK Yu Y Wulamu S Wang Y Wang Q Zhou Y. Structural Analysis of Water-Soluble Polysaccharides Isolated From Panax Notoginseng. Int J Biol Macromol (2020) 155:376–85. doi: 10.1016/j.ijbiomac.2020.03.233 32240740

[26] Yang X Wu Y Zhang C Fu S Zhang J Fu C. Extraction, Structural Characterization, and Immunoregulatory Effect of a Polysaccharide Fraction From Radix Aconiti Lateralis Preparata (Fuzi). Int J Biol Macromol (2020) 143:314–24. doi: 10.1016/j.ijbiomac.2019.11.208 31786293

[27] Liu X Liu D Chen Y Zhong R Gao L Yang C. Physicochemical Characterization of a Polysaccharide From Agrocybe Aegirita and its Anti-Ageing Activity. Carbohydr Polymers (2020) 236:116056. doi: 10.1016/j.carbpol.2020.116056 32172871

[28] Zhang X Yu L Bi H Li X Ni W Han H. Total Fractionation and Characterization of the Water-Soluble Polysaccharides Isolated From Panax Ginseng C. A. Meyer. Carbohydr Polymers (2009) 77:544–52. doi: 10.1016/j.carbpol.2009.01.034

[29] Jiao L Zhang X Wang M Li B Liu Z Liu S. Chemical and Antihyperglycemic Activity Changes of Ginseng Pectin Induced by Heat Processing. Carbohydr Polymers (2014) 114:567–73. doi: 10.1016/j.carbpol.2014.08.018 25263928

[30] Li Ji ZJ Ying X Yue Q Zhou Y Sun L. Structural Characterization of Alkali-Soluble Polysaccharides From Panax Ginseng C. A. Meyer. R Soc Chem (2018) 5:171644. doi: 10.1098/rsos.171644 PMC5882694 29657770

[31] Herbst RS Morgensztern D Boshoff C. The Biology and Management of non-Small Cell Lung Cancer. Nature (2018) 553:446–54. doi: 10.1038/nature25183 29364287

[32] Yu L Zhang X Li S Liu X Sun L Liu H. Rhamnogalacturonan I Domains From Ginseng Pectin. Carbohydr Polymers (2010) 79:811–7. doi: 10.1016/j.carbpol.2009.08.028

[33] Inngjerdingen KT Patel TR Chen X Kenne L Allen S Morris GA. Immunological and Structural Properties of a Pectic Polymer From Glinus Oppositifolius. Glycobiology (2007) 17:1299–310. doi: 10.1093/glycob/cwm088 17726087

